# Molecular Recognition of H3/H4 Histone Tails by the Tudor Domains of JMJD2A: A Comparative Molecular Dynamics Simulations Study

**DOI:** 10.1371/journal.pone.0014765

**Published:** 2011-03-25

**Authors:** Musa Ozboyaci, Attila Gursoy, Burak Erman, Ozlem Keskin

**Affiliations:** Center for Computational Biology and Bioinformatics and College of Engineering, Koc University, Istanbul, Turkey; German Cancer Research Center, Germany

## Abstract

**Background:**

Histone demethylase, JMJD2A, specifically recognizes and binds to methylated lysine residues at histone H3 and H4 tails (especially trimethylated H3K4 (H3K4me3), trimethylated H3K9 (H3K9me3) and di,trimethylated H4K20 (H4K20me2, H4K20me3)) via its tandem tudor domains. Crystal structures of JMJD2A-tudor binding to H3K4me3 and H4K20me3 peptides are available whereas the others are not. Complete picture of the recognition of the four histone peptides by the tandem tudor domains yet remains to be clarified.

**Methodology/Principal Findings:**

We report a detailed molecular dynamics simulation and binding energy analysis of the recognition of JMJD2A-tudor with four different histone tails. 25 ns fully unrestrained molecular dynamics simulations are carried out for each of the bound and free structures. We investigate the important hydrogen bonds and electrostatic interactions between the tudor domains and the peptide molecules and identify the critical residues that stabilize the complexes. Our binding free energy calculations show that H4K20me2 and H3K9me3 peptides have the highest and lowest affinity to JMJD2A-tudor, respectively. We also show that H4K20me2 peptide adopts the same binding mode with H4K20me3 peptide, and H3K9me3 peptide adopts the same binding mode with H3K4me3 peptide. Decomposition of the enthalpic and the entropic contributions to the binding free energies indicate that the recognition of the histone peptides is mainly driven by favourable van der Waals interactions. Residue decomposition of the binding free energies with backbone and side chain contributions as well as their energetic constituents identify the hotspots in the binding interface of the structures.

**Conclusion:**

Energetic investigations of the four complexes suggest that many of the residues involved in the interactions are common. However, we found two receptor residues that were related to selective binding of the H3 and H4 ligands. Modifications or mutations on one of these residues can selectively alter the recognition of the H3 tails or the H4 tails.

## Introduction

Histone methylation and demethylation have significant roles in transcriptional regulation and chromatin condensation [1]. Methylation of lysine residues in H3 and H4 histone proteins are specifically involved in activation or repression of specific genes [2], [3], [4], [5]. These histone proteins are one of the most slowly evolving proteins among all eukaryotic proteins and are extremely conserved [6] (also see [7] in all species). It was first hypothesized that methylation of lysine residues on histone molecules were irreversible and could be replaced by a new methyl-free histone molecule to erase the methyl mark [8], [9], [10]. However, recent studies show that the histone lysine methylation is not irreversible and histone lysine demethylases (HDMs) are employed for the removal of the methyl marks from the lysine residues of the histones [11], [12].

JMJD2A, a histone lysine demethylase, catalyses the demethylation reaction of di- and tri-methylated Lys9 and Lys36 of H3 tail [8], [13]. The JMJD2A protein consists of four different domains: JmjC, JmjN, 2 PHD and 2 tandem tudor domains. The catalytic site of the enzyme is composed of JmjC and JmjN domains. Tudor domains of JMJD2A bind mostly to trimethylated H3K4, trimethylated H3K9 and di,trimethylated H4K20 [14]. In mammals, methylation of H3K4 is mostly associated with transcriptional activation, antagonizing the effect of the methylation of H3K9 and H3K36 whereas methylation of H4K20 is associated with gene silencing [15]. Demethylation reaction can result in both silencing and activation of gene transcription. Since JMJD2A enzymes function mostly in multimeric forms, different combinations of interactions with methylated H3K4, H3K9 and H4K20 might target the enzymes to different destinations [16].

The tudor domains interact with different histone tails by different binding modes. It was shown that specific point mutations on these domains repress specific recognition of one tail but not the others [16]. It is of great importance to understand the underlying specificity of the recognition of the different histone tails by the tudor domains to design selective drugs for targeting the tudor domains.

**Figure 1:**
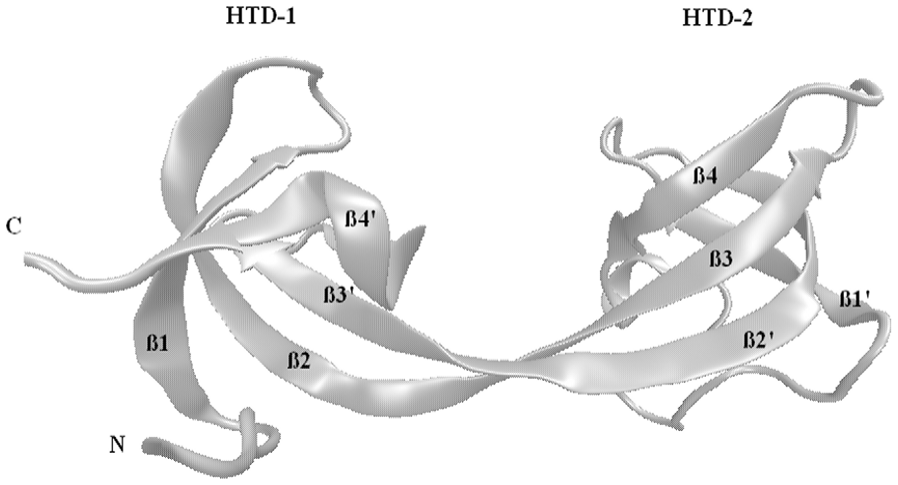
Secondary structure of JMJD2A-tudor domains. The tandem hybrid tudor domains have an interdigitated structure in which structural motifs are exchanged between each other. β2 and β3 strands are swapped between the hybrid domains. The two lobes of the structure are named as Hybrid tudor Domain 1 (HTD-1) and Hybrid tudor Domain 2 (HTD-2).

As illustrated in Figure 1, the double tudor domains of JMJD2A are tandem and bilobal. The tandem domains have a saddle shaped structure in which each lobe interweaves with each other [16]. Lobes in the tandem tudor domains are named as hybrid tudor domain 1 and 2 (HTD-1,2). Methylated peptide only binds to a specific crevice of HTD-2 [16]. HTD-2 is more negatively charged compared to HTD-1 on the surface [17]. This might facilitate the binding of the positively charged methylated peptides. Previously, structures of methylated peptides (H3K4me3 and H4K20me3) interacting with JMJD2A-tudor were reported [16], [17]. These two H3 and H4 tails do not share any sequence similarity but methylated lysine residues. Comparison of the two crystal structures, H3K4me3-JMJD2A-tudor and H4K20me3-JMJD2A-tudor, indicates that the tails have different binding modes and adapt opposite orientations [16], [17]. Furthermore, the experimental studies identified the residues that play critical roles in complex formation. Although many of the interacting residues were identified in these two complexes, why they bind in different orientations is still not well understood.

Dynamics of the two tails might be important in adapting different binding modes. This is the first study, to our knowledge, looking at the dynamic aspects to get an insight on the recognition and binding of JMJD2A to different histone tails. We have studied four complexes: JMJD2A-tudor domain structures bound to i) H3K4me3, ii) H4K20me3, iii) H4K20me2 and iv) H3K9me3. Since the structures of the last two complexes are not available experimentally, we first modelled the bound complexes. Molecular dynamics simulations of JMJD2A-tudor liganded to H3K4me3, H4K20me3, H4K20me2 and H3K9me3, as well as the free tudor domain and the free peptide ligands were performed for 25 ns. Binding free energies and critical residues were calculated by the molecular mechanics Poisson Boltzmann surface area (MM-PBSA) and molecular mechanics generalized Born surface area (MM-GBSA), respectively. We show that the binding mode of H3K9me3 is the same as that of H3K4me3; further many of the residues involved in recognition of these two peptides are common. We identify the important interactions between the tudor domains and the peptides. We find that Ser938 and Glu929 of JMJD2A-tudor are involved in strong interactions with H4 and H3 peptides, respectively. Along with residues Asp945, Asn940 and Asp939 of the protein, we determine new critical residues (hot spots) such as Ser936, Phe937 and Asp969. We further find that some hot spots are used in both binding to H3 and H4 tails, whereas some other hot spots are specific to the tail type. So, these residues might be important for the specificity JMJD2A-tudor to bind to different histone tails.

## Results

### Molecular motions of JMJD2A-tudor

**Figure 2:**
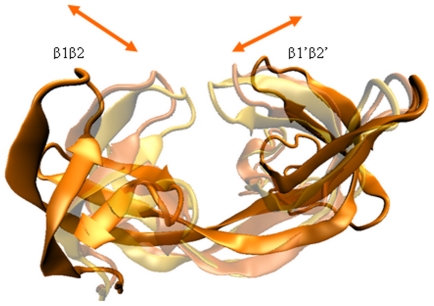
Motion between the tandem hybrid tudor domains of JMJD2A. The arrows show the direction of opening/closing motion. Different colours indicate the conformations of different snapshots during the simulations.

We observed that HTD-1 and HTD-2 parts periodically undergo a swing-like motion (Figure 2) (determined by the change in the radius of gyration values, shown in Figure S1, and visually investigating the trajectories). The periodicity was varying for each of the structures with different tails. This motion was highly dominant in the structure where there is no bound histone tail. This motion should be critical for association/dissociation of the tudor domains and histone tails since the tails bind at the β1′β2′ and β3β4 flap regions in HTD-2. The RMSD values of the tudor domains were stable, although proteins underwent large conformational changes (Figure S2). When the protein was bound to the histone tail, the change in the structural shape had a lower frequency proposing that binding has an important role in the global motions of the protein. We analyzed the distance between the tip of a flap region and the centre of the protein, we obtained a periodicity of around 10–12 ns for this motion (Figure S3). The periodicity of the bound tudor domains is lower. Therefore, the bound histone tails change the global motion of the tudor domains. We suggest that the faster opening-closing motion of the tudors increases the possibility for searching the proper orientation of the tails to bind to the tudor domain flap region.

### Binding Site Differences

**Figure 3:**
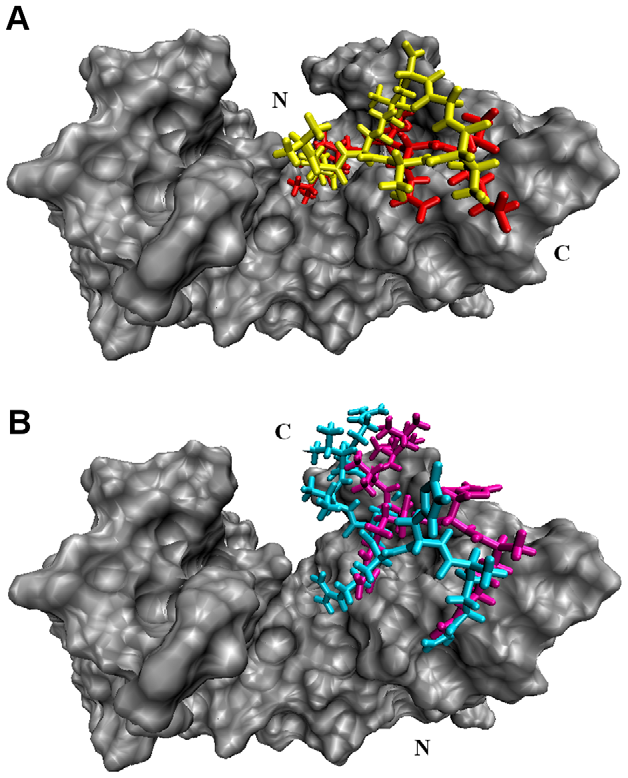
Two different binding modes of JMJD2A-tudor (A) with liganded to H3K4me3 (red) and H3K9me3 (yellow) (B) with liganded to H4K20me3 (cyan) and H4K20me2 (magenta). First two peptides bind to the HTD-2 with a similar mode in the same orientation, whereas the later peptides bind in the opposite orientation with a similar mode.

H3K4me3 and H4K20me3 peptides were shown to bind to JMJD2A-tudor domain in two distinct modes in a previous study [16]. In this study, we observed that no additional binding modes but these two were adopted for the recognition of the H4K20me2 and H3K9me3 peptides as well. The two different binding modes [Figure 3 (A,B)] were distinguished by the orientations of the peptides which were located in the opposite directions relative to each other which might be seen in other protein complexes.[18] H3 and H4 interact with different residues located on JMJD2A-tudor domain. The first binding mode is adopted by the H3 peptides, whereas the other mode is adopted by the H4 peptides. H4K20me3 and H4K20me2 adopted the same binding mode, not surprisingly, since the starting structures are the same but the methylated lysine residues are different. As observed from the experimental data; on the other hand, H3K4me3 adopts a different binding mode[16]. The same binding mode is also adopted by H3K9me3 peptide starting from an independent docking simulation. Interactions between the receptor molecule and the two H3 peptides are alike; thus showing that H3K9me3 and H3K4me3 are recognized by JMJD2A-tudor with a similar fashion.

**Figure 4:**
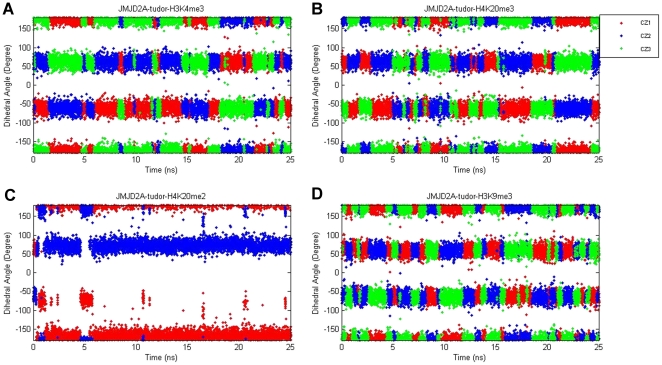
Dihedral angles of the methylated lysine residue defined by C_δ_, C_ε_, N_ζ_ and CZ atoms in the JMJD2A-tudor molecule liganded to H3K4me3 (A), H4K20me3 (B), H4K20me2 (C) and H3K9me3 (D), respectively.

Consistent with the experimental data, methylated lysine residues of the four peptides were caged by the aromatic side chains of Phe932, Trp967 and Tyr973 of JMJD2A-tudor HTD-2 throughout the simulations. In this aromatic cage, methyl groups of the trimethyllysine residues were observed to be rotating freely, whereas the methyl groups of the dimethyllysine residue were stable during the molecular dynamics simulations. To understand the basis of this behaviour, time evolution of the torsion angles defined by C_δ_, C_ε_, N_ζ_ and CZ atoms of the methylated lysine residues were investigated. Figure 4 shows the possible rotameric states of the methyl groups throughout the simulations. The bands correspond to populated rotamers. As illustrated in Figure 4 (A, B and D), there are three equally probable states for each of the methyl groups in trimethylated lysine residues. Conversely, methyl groups of the dimethylated structure [see Figure 4C] show a distinct fluctuation pattern. The defined torsion angles of the trimethylated residues were mostly oscillating at the gauche^+^ (g^+^) states around 60 degrees, at the gauche^−^ (g^−^) states around −60 degrees and at the trans (t) states around 180 degrees; whereas the angles of the dimethylated lysine were mostly oscillating at the gauche^+^ (g^+^) and the trans (t) states. This observation indicates that the methyl groups in the methylated lysine residue fluctuate about two or three conformations depending on the number of methyl groups. More intriguingly, conformations of these subgroups shifted continuously by rotating throughout the simulation for the trimethylated lysine residues; while the methyl groups in the dimethylated lysine residue retained their conformations throughout the simulation.

**Figure 5:**
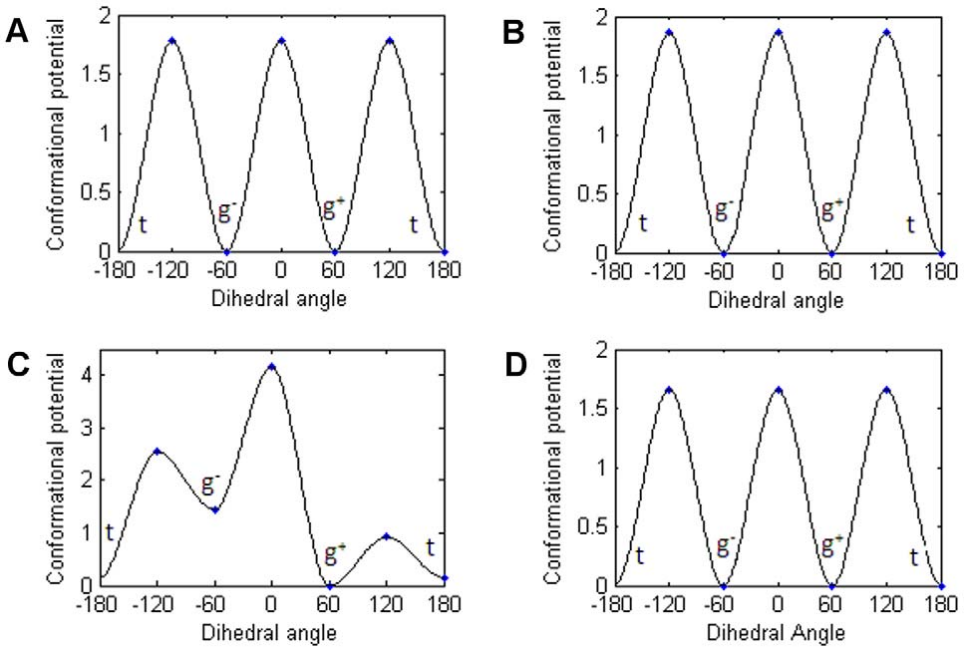
Activation potential energies between conformational states of the methylated lysine residues of H3K4me3 (A), H4K20me3 (B), H4K20me2 (C) and H3K9me3 (D) peptides. Gauche^+^, gauche^−^ and trans conformations were shown as g^+^, g^−^ and t respectively. Energy values are in kcal/mol units and a temperature dependant c constant was removed from each of the energy values.

To seek the required conformational potentials, activation energies between each conformational state [Table 1] were calculated (see methods for the details of the calculations). Figure 5 shows the torsional bond energy profile of the methyl group. The x-axes represent the torsional angle range [−180, 180]. The three possible states (g^+^, g^−^, t) and their corresponding energy values can be depicted from the figures. As seen in Figure 5 (A,B and D), trimethylated residues have the same energy barriers in transitions between the three states, whereas the dimethylated residue has differing energy barriers [Figure 5C]. Comparing the energy values of the systems showed that activation energy barriers of the methyl conformations are highest for H4K20me2 and almost the same for the rest of the trimethylated residues. In this respect, it is more likely for the trimethylated lysine residue of H3K9me3 to change its conformation more freely than the dimethylated residue. Additionally, in this study we also found that methyl groups of the dimethyl lysine of H4K20 peptide were mostly in g^+^ and t states and transitions to other states were observed to be highly unfavourable energetically.

**Table 1:** Activation energy barriers between conformations.

	g^+^→g^−^	g^−^→g^+^	g^+^→t	t→g^+^	g^−^→t	t→g^−^
**H3K4me3**	1.8	1.8	1.8	1.8	1.8	1.8
**H4K20me3**	1.9	1.9	1.9	1.9	1.9	1.9
**H4K20me2**	4.2	2.7	0.9	0.8	1.1	2.4
**H3K9me3**	1.7	1.7	1.7	1.7	1.7	1.7

All values are in kcal/mol units and are with respect to an arbitrary datum line.

### Critical Interactions

**Figure 6:**
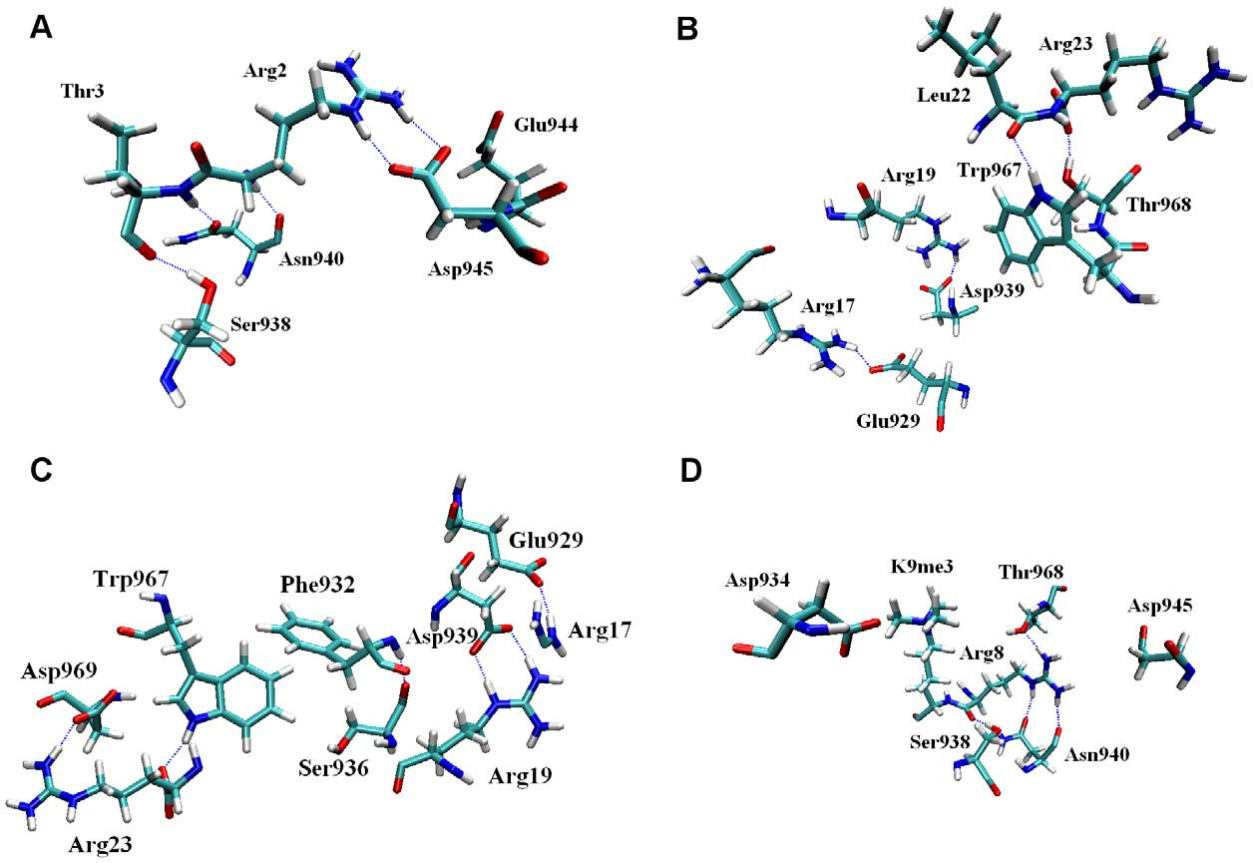
Detailed views of the residues that are involved in forming hydrogen bonds and salt bridges in the tudor domains liganded to H3K4me3 (A), H4K20me3 (B), H4K20me2 (C) and H3K9me3 (D) structures. Hydrogen bonds are represented in blue dashed lines.

Hydrogen bonds and salt bridges are important indicators of a stable complex structure in which recognition of the constituents is achieved with high affinity. To elucidate the critical interactions between JMJD2A-tudor and the histone peptides, we analyzed the hydrogen bonds and the salt bridges that were formed during the molecular dynamics simulations. High occupancy hydrogen bonds and electrostatic interactions were observed throughout the trajectory (see Table 2 for a list of residues involved in H-bonding and electrostatic interactions). These interactions are highlighted in Figure 6 for the four complexes. Interactions obtained from molecular dynamics simulations should be important and have a significant role in understanding the stability of different histone peptides by the tudor domains.

**Table 2:** The residues involved in forming hydrogen bonds and salt bridges based on molecular dynamics simulations.

Residue at JMJD2A	H3K4me3	H4K20me3	H4K20me2	H3K9me3
Glu929		Arg17	Arg17	
Phe932		Arg17		
Asp934			K20me2	K9me3
Ser938	Thr3			Arg8
Asp939	Gln5	Arg19	Arg19	
Asn940	Thr3			Arg8
Glu944	Arg2			
Asp945	Arg2			Arg8
Trp967		Leu22,Arg23	Arg23	
Thr968		Arg23		Arg8
Asp969		Arg23		

We observed that, structures of JMJD2A-tudor liganded to H3K4me3 and H4K20me3 peptides obtained via molecular dynamics simulations were in good agreement with experimental studies [16], [17] In the JMJD2A-tudor binding to H3K4me3 structure [see Figure 6A], Asp945 was reported to be one of the most important residues in ligand binding by interacting with Arg2 of the histone tail [16]. Indeed, together with Glu944, Asp945 interacted with Arg2 of histone tail with a high occupancy in molecular dynamics simulation. Moreover, OD1 and OD2 atoms of Asp945 formed salt bridges with NH2 and NE atoms of H3K4me3 Arg2 during the whole simulation. Asp945 in other complexes; however, was not observed to be interacting with the ligand peptides. Instead, Asp945 formed hydrogen bonds and electrostatic interactions with Arg966 of JMJD2A-tudor. In experimental studies, Asn940 was also found to be very important in the recognition of H3K4me3. Supporting the experimental studies, in molecular dynamics simulations, Asn940 interacted with Thr3 of the H3 tail with occupancy of 12.78% throughout the simulation. As expected from the previous ITC experiments, Asn940 did not interact with H4K20me3 and H4K20me2 peptides. The hydrogen atoms on NH1 and NH2 of Arg8 on H3K9me3, which play crucial role in binding, formed hydrogen bonds with Asn940. In the H4K20me3-tudor domain complex [see Figure 6B], Asp939 was proposed to be highly important. In the molecular dynamics simulations, Asp939 formed hydrogen bonds and salt bridges with H4K20me3 Arg19, consistent with previous studies. Interestingly, Arg19 of H4K20me2 formed unstable hydrogen bonds with Asp939, suggesting that Arg19 of H4K20me2 has less importance than Arg19 of H4K20me3. Gln5 of H3K4me3 was also observed to be interacting with Asp939 in the first 2.5 ns and between 7.5–15 ns of the simulation via weak hydrogen bonds. Another significant interaction was observed between Ser938 of the receptor and the peptide residues Thr3 of H3K4me3 and Arg8 of H3K9me3. The later interactions suggest that binding of H3K4me3 and H3K9me3 tails by the tudor domains are similar.

A remarkably strong hydrogen bond and coulombic interactions occurred between Glu929 and Arg17 in the last 20 ns of the JMJD2A-tudor-H4K20me3 MD simulation. With a high occupancy, HH12 atom on NH1 and HH22 atom on NH2 of Arg17 were hydrogen bonded to OE1 and OE2 atoms of Glu929, whereas in JMJD2A-tudor H4K20me2 structures, Arg17 was observed to interact with two other residues. Arg17 of H4K20me2 formed hydrogen bonds with the backbone oxygen atom of Phe932 and the backbone oxygen atom of Ser936. The interactions with these residues were observed in the first 12 ns of the simulation with high occupancies, while hydrogen bond and salt bridge interactions with Glu929 came within the last 10 ns of the simulation proposing that Glu929 may not have much significance in binding to H4K20me2. Another strong hydrogen bond interaction appeared between the backbone oxygen atom of Leu22 of H4K20me3 and HE1 atom on NE1 of Trp967. This hydrogen bond had 35.16% occupancy and was consistent throughout the simulation. Trp967 also formed hydrogen bonds with the terminal oxygen and the backbone oxygen atoms of H4K20me3 Arg23 with less occupancy compared to the first one. Like the tudor binding to H4K20me3 structure, Trp967 was observed to interact with the backbone oxygen atom of Arg23 of H4K20me2 via a strong hydrogen bond. Unlike the trimethylated structure, this bond was permanent and had a high occupation of 76.90%. Arg23 of H4K20me2 also formed a hydrogen bond with OD1 atom of Asp969, in the last 15 ns of the simulation. Furthermore, during molecular dynamics simulations, salt bridges between Asp969 and Arg23 were observed in both H4K20me3 and H4K20me2 structures. Supporting the experimental data, Tyr942 and Thr968 did not form any significant interactions with the methylated histone tails.

### Free Energy Decomposition of JMJD2A-tudor-histone tail complexes

#### Enthalpy calculations

Change in enthalpy upon complexation of JMJD2A-tudor with the peptide tails was calculated by MM-PBSA method. Contributions to the binding free energies were decomposed into its components [see Table 3]. Non polar and internal energy contributions, which come from the sum of bond, angle and dihedral energies, constitute a small part of the enthalpy. As expected, the electrostatic and the van der Waals obtained from the MM part and the polar contribution obtained from the PB calculations constitute the major part of the enthalpy.

**Table 3:** Free energy contributions coming from molecular mechanics and PB calculations.

Complex	ΔE^ele^	ΔE^vdw^	ΔE^int^	ΔE^gas^	ΔG^SA^	ΔG^PB^	ΔG^PBSA^	ΔG^PB^+ ΔE^ele^	ΔG^MMPBSA^
**H3K4me3**	−108,36 (0,59)	−39,65 (0,53)	4,2 (1,08)	−143,81 (1,24)	−5,66 (0,03)	109,95 (0,44)	104,29 (0,43)	1,58 (0,35)	−39,52 (1,14)
**H4K20me3**	−206,84 (0,57)	−45,81 (0,54)	−6,07 (1,1)	−258,72 (1,25)	−6,44 (0,04)	219 (0,42)	212,56 (0,41)	12,16 (0,38)	−46,16 (1,18)
**H4K20me2**	−229,76 (0,52)	−44,44 (0,53)	−4,21 (1,12)	−278,42 (1,26)	−6,31 (0,03)	228,72 (0,41)	222,42 (0,41)	−1,04 (0,33)	−56 (1,17)
**H3K9me3**	−110,83 (0,55)	−40,31 (0,53)	9,63 (1,1)	−141,51 (1,28)	−6,04 (0,03)	111,28 (0,46)	105,25 (0,45)	0,46 (0,38)	−36,27 (1,19)

All values in the table are in kcal/mol unit. Standard errors of corresponding values are given in parentheses.

For each structure, mean values of the contributions for different dielectric constants are represented.

In all of the four complexes, intermolecular coulombic forces and van der Waals interactions favour ligand binding. Internal energies also favour binding of H4K20me2/3 ligands, whereas disfavour binding of H3K4me3 and H3K9me3 ligands proposing that the conformational changes upon binding lead to internal strains in JMJD2A tudor- H3K4me3/H3K9me3 complexes [19], [20]. The nonpolar solvation free energy values for the PB model, which was obtained via solvent accessible surface area (SASA) calculations, contributed favourably to the total binding free energy in four of the complexes. The polar contributions to the solvation free energy for the PB model, on the other hand, considerably disfavoured the binding for all complexes. The total electrostatic energies (ΔE*^ele^*+ΔG*^PB^*) are positive in the tudor-H3K4me3/H3K9me3 and H4K20me3 complexes, indicating that overall coulombic forces disfavour binding, whereas the total electrostatic energy is negative in the tudor-H4K20me2 complex implying that the total coulombic interactions slightly favour binding. The compensation of the electrostatic energies with the polar solvation free energies lean to the high cost of desolvation of the uncounterbalanced polar and charged groups upon complex formation. Overall, this proposes that, for all complexes binding was mainly driven by favourable van der Waals interactions. The non polar contributions to the total solvation free energy and the molecular mechanical internal energies have a less significant contribution to the binding.

#### Entropy Calculations

The continuum solvent models estimate the free energy comprising the contribution of the solvent entropies. The entropic contributions [Table 4] result from the conformational changes in rotational, translational and vibrational degrees of freedom of solute upon complex formation. The loss in translational and rotational degrees of freedom was calculated based on classical statistical mechanics; whereas, the loss in vibrational degrees of freedom was calculated using normal mode analysis. Standard errors of the entropic contributions entirely arose from the vibrational degrees of freedom by around 1kcal/mol which is highly reasonable in terms of internal accuracy of the snapshots.

**Table 4:** Entropy contributions of the structures.

Complex	−TΔS_trans_	−TΔS_rot_	−TΔS_vib_	−TΔS_tot_
**H3K4me3**	13,73 (0)	12,06 (0,01)	0,28 (1,05)	26,08 (1,05)
**H4K20me3**	13,91 (0)	12,55 (0,02)	5,97 (1,12)	32,43 (1,12)
**H4K20me2**	13,9 (0)	12,63 (0,01)	9,76 (1,07)	36,29 (1,07)
**H3K9me3**	13,62 (0)	11,93 (0,01)	3,72 (1,1)	29,27 (1,1)

All values are in kcal/mol units. Standard errors of corresponding values are given in parentheses.

#### Binding Free Energies

The sum of entropic and enthalpic contributions gives the binding free energy. Because there are experimental data for only two of the four complexes, our comparison with experimental data involves only these two of the interactions. In this respect, discussions in model comparisons are based on the available data in this study. Calculated binding free energies may deviate from the experimental values owing to the omitted contributions of enthalpy and entropy. These contributions are: configurational entropy of the side chains which might be significant depending on the structure, the dielectric constant, the bond radii and the model chosen for solving the solvation free energy. PB binding free energies for H4K20me3 and H3K4me3 are very close to each other (−13.73 and −13.33kcal/mol, respectively). Experimental dissociation constants available for these two complexes are also very close to each other [see Table 5]. We did not convert the dissociation constants to free energies since we do not know the standard-state concentration in the experiments.

**Table 5:** Binding free energy components of the structures calculated from PB method and experimental disassociation constants.

Complex	ΔG^MMPBSA^	−TΔS_tot_	ΔG_PB_	K_d,exp_
**H3K4me3**	−39,52	26,08	−13,44	0.50±0.03
**H4K20me3**	−46,16	32,43	−13,73	0.40±0.03
**H4K20me2**	−56	36,29	−19,71	n/a
**H3K9me3**	−36,27	29,27	−7	n/a

All values are in kcal/mol units.

Enthalpic and entropic calculations were performed based on multiple trajectory approach.

ΔG_PB_ values show that JMJD2A-tudor-H4K20me2 complex is the most favourable one with a distinct binding free energy of −19,71 kcal/mol [Table 5]. Following that, JMJD2A-tudor-H4K20me3 and JMJD2A-tudor-H3K4me3 structures appear with binding free energies of −13,73 kcal/mol and −13.44 kcal/mol respectively. JMJD2A-tudor-H3K9me3 has the least favourable interaction compared to the other three structures with a binding free energy of −7.00 kcal/mol.

### Hot Spots in the Interfaces of Tandem Tudor Domains of JMJD2A and H3/H4 Tails

**Figure 7:**
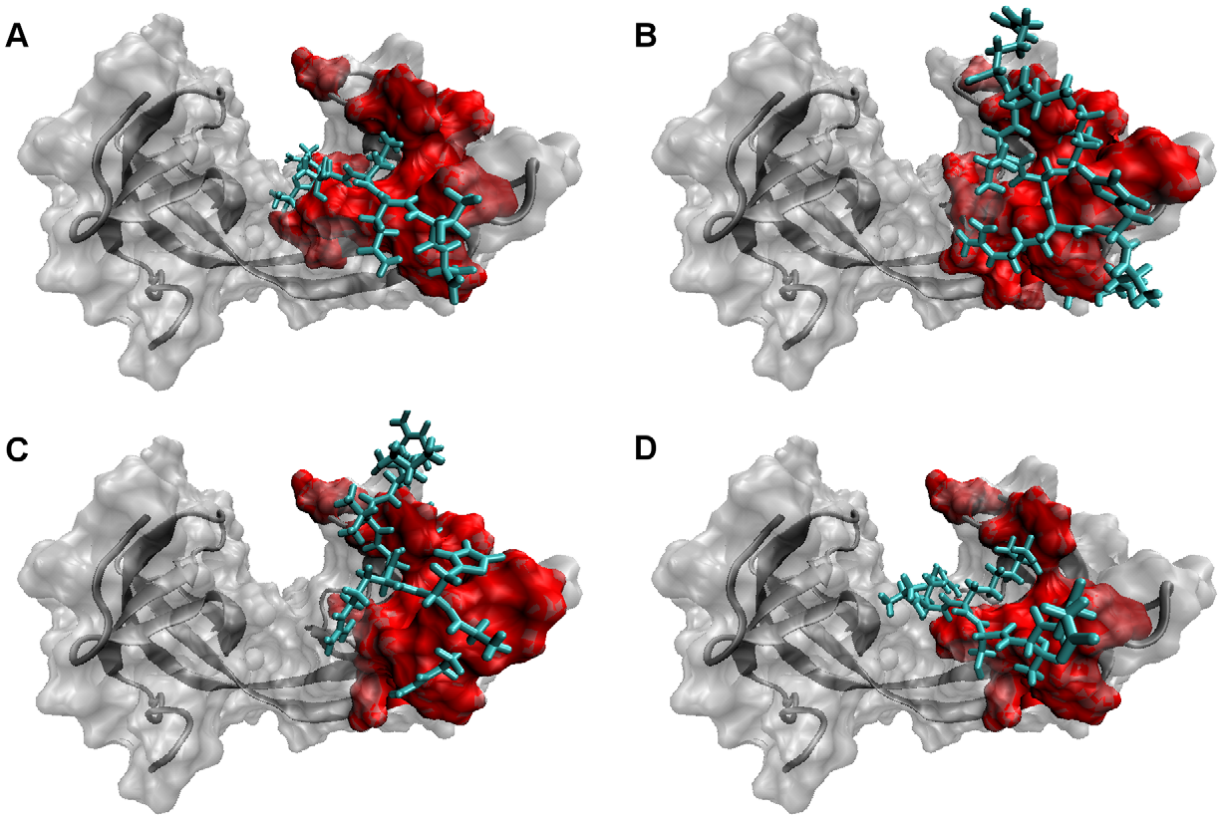
Molecular surface representation of JMJD2A-tudor. The hotspot residues in the receptor are shown with red colour. Shown in licorice representation with cyan colour, the ligand residues of H3K4me3 (A), H4K20me3 (B), H4K20me2 (C) and H3K9me3 (D) structures are represented in the figure.

Hot spots are important in determining the binding affinities [21], [22], [23], [24]. In this study, MM-GBSA approach was used to find the critical residues (hot spots) taking role in complex formation. To accomplish that, the enthalpic contribution to the binding free energy was decomposed into its residual components and the residual components were decomposed into pair-wise components. Based on the contribution of residues to the binding free energy difference, the ones having significance in binding were identified [Table 6]. As shown in Figure 7, the residues in the ligand and in HTD-2 of the receptor which have a contribution of more than absolute 1.0 (kcal/mol) to the enthalpic contribution the total binding free energy difference were defined as hotspots. Since the extraction of the entropic contribution per-residue from the binding free energy was not available, hot spots were determined on the basis of the enthalpy terms.

**Table 6:** Hotspots of the structures with corresponding total energetic contributions and the side chain energetic contributions shown in parentheses.

	Residue	H3K4me3	H4K20me3	H4K20me2	H3K9me3
JMJD2ATudor	Phe927	−1,92 (−1,39)	−1,21 (−1,00)	−1,61 (−1,24)	
	Glu929		−1,69 (−1,31)	−0,86 (−0,70)	
	Phe932	−3,09 (−2,78)	−2,53 (−2,36)	−2,56 (−2,05)	−2,96 (−2,63)
	Ser936	−2,31 (−1,17)	−2,32 (−1,23)	−3,27 (−1,54)	−2,99 (−1,53)
	Phe937	−3,29 (−1,52)	−4,76 (−3,32)	−4,36 (−2,47)	−3,18 (−1,27)
	Ser938	−1,66 (−1,01)	−1,58 (−0,74)	−1,24 (−0,75)	
	Asp939		−1,35 (−1,01)	−1,04 (−0,79)	
	Asn940	−3,88 (−2,58)			−2,72 (−2,07)
	Leu941	−2,69 (−1,21)	−1,29 (−0,66)		−1,88 (−0,75)
	Asp945	−1,33 (−1,18)	−1,07 (−0,85)		
	Trp967	−3,61 (−3,06)	−4,93 (−3,95)	−5 (−3,66)	−3,8 (−3,53)
	Asp969	−1,92 (−0,95)	−2,86 (−1,48)	−2,93 (−1,76)	−1,88 (−0,54)
	Tyr973	−1,06 (−1,59)	−1,3 (−1,71)	−1,54 (−2,04)	
H3K4	Arg2	−3,28 (−4,18)			
	Thr3	−2,94 (−1,17)			
	K4me3	−9,95 (−8,50)			
	Gln5	−2,65 (−0,95)			
H4K20	Arg17		−5,12 (−4,81)	−6,33 (−5,86)	
	His18		−1,57 (−0,20)	−3,27 (−1,89)	
	Arg19		7,17 (−4,91)	−4,8 (−3,19)	
	K20me2/3		−9,47 (−8,99)	−11,25 (−10,06)	
	Arg23		−2,31 (0,54)	−2,67 (−0,36)	
H3K9	Arg8				−5,84 (−5,03)
	K9me3				−10,68 (−9,34)
	Ser10				−1,04 (−0,17)

All values are in kcal/mol units.

Contributions to the overall binding free energies of Phe932, Trp967 and Tyr973, neighbouring the methylated residues, were found to be significant for all complexes. The residues formed strong van der Waals interactions with trimethyllysine residue via their aromatic side chains. The only exception was that the relatively high value of Tyr973 of JMJD2A-tudor-H3K9me3 structure (−0.6 kcal/mol). This energy of Tyr973 arose from the slightly less favourable interactions with Asp933, Asp934 and Trp967. However, it should be noted that there was a highly favourable interaction between this Tyr973 and K9me3 (−2 kcal/mol, data not shown) indicating that Tyr973 was crucially involved in binding of the trimethyllysine residue to the receptor, although it did not appear as a hotspot in the list. Asp969, another significant residue in recognition of the peptides, formed van der Waals interactions with Trp967 and electrostatic interactions with Gln971 upon complexation, hence favouring the binding. Ser936 and Phe937 of JMJD2A-tudor were also found to be significant in all of the four structures, in terms their energetic contributions to the enthalpy upon complex formation. These two residues were involved in many favourable van der Waals interactions with methylated lysine residues along with Gln5 of H3K4me3, His18 of H4K20me2/3 and Ser10 of H3K9me3. Thus Ser936 and Phe937 appeared to be vital in complex formation.

**Figure 8:**
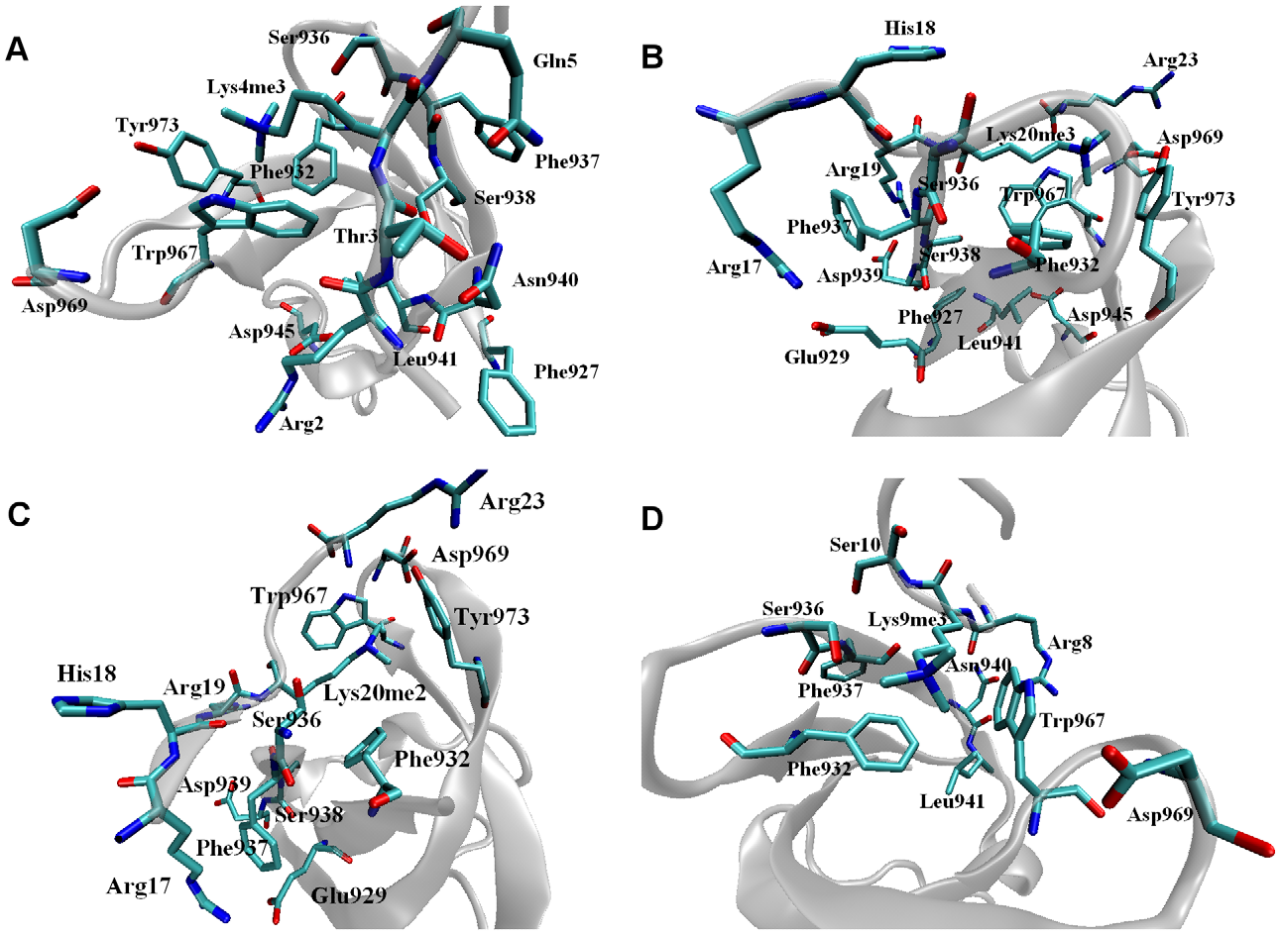
Snapshots of the MD simulations showing the hotspot residues of JMJD2A-tudor complexed with H3K4me3 (A), H4K20me3 (B), H4K20me2(C) and H3K9me3 (D) peptides.

Asn940 is found to have the highest binding free energy contribution (–3,88 kcal/mol [Table 6]) between JMJD2A-tudor and H3K4me3 histone tail. The high contribution to the overall favourability is mainly driven by van der Waals and electrostatic interactions between Asn940 and Ala1, Arg2 and Thr3 of the histone ligand. Interactions with Arg2 of the peptide and Leu941 [Figure 8A] of JMJD2A-tudor also favour binding with a high contribution to the total free energy of binding. Asn940 which is similar to Leu941 in binding to H3K4me3 also had favourable interactions with Arg8 of H3K9me3 [Figure 8D]. Furthermore, Leu941 favoured binding to H4K20me3 peptide by forming favourable interactions with the receptor residues. Interestingly contribution of internal energies to the free energy was very significant for Leu941, this indicates that final conformation of Leu941 is more favourable upon binding to H3K4me3 and H3K9me3 peptides.

Asp939 is known to be very important in complex formation of JMJD2A-tudor-H4K20me3, we also observed a binding free energy of −1,35 kcal/mol [Table 6]. Further investigation suggested that this residue had a significant role in complexation by favouring the binding of Arg17 and Arg19 of H4K20me2 and H4K20me3 peptides [Figures 8B and 8C]. Favourability of Asp939 was driven by electrostatic interactions which comprised of the hydrogen bonds and salt bridges, even though a large portion of the electrostatic term was cancelled by the desolvation penalty in both of the H4 tails.

Glu929 contributed to the enthalpic binding free energies of H4K20me3 and H4K20me2 by values of −1.69 kcal/mol and −0.86 kcal/mol respectively, which were dominated by the electrostatic contribution coming mostly from the hydrogen bond and coulombic interactions with Arg17 in the ligand. It should be noted that Glu929 especially was very significant in the binding to the trimethylated H4K20 peptide, since very low energetic values came from Arg17.

Strikingly, Asp945 [Table 6 and Figure 8A] displayed a favourable interaction (−1,33 kcal/mol) upon complex formation with H3K4me3 ligand, and the residue was known to be very crucial in binding to the ligand. Further, dominated by side chain electrostatic interactions, the notable contribution to the free energy difference of Arg2 in the histone tail suggests that Asp945 has a major role in binding, in spite of the fact that the high desolvation penalty cancels the overall electrostatic contribution term. Although Asp945 [Figure 8B] was defined as a hotspot in the recognition of H4K20me3 peptide in this study, energetic contributions of this residue were not arisen from the interactions with the peptide ligand. The contributions were mainly supplied through side chain van der Waals forces with the receptor residues.

Unlike the recognition of trimethylated H4K20 peptide, Asp934 and Gly935, which we designated as hotspots, were employed in the complexation of the dimethylated H4K20 peptide with JMJD2A-tudor protein. Asp934 had favourable interactions with the dimethyllysine that were mainly dominated by hydrogen bonds. Asp934 interacted with the trimethyllysine in the H4K20 ligand without forming hydrogen bonds; therefore the resulting energetic value is lower for the dimethylated structure. Arg17 of the ligand contributed to the free energy difference of Gly935 considerably by attractive van der Waals and electrostatic interactions; hence emphasizing the role on binding to the receptor. Together with Arg17, Gly935 was also occupied in van der Waals interactions with His18 of H4K20me2.

## Discussion

Methylation marks on histone tails are of great importance in transcriptional regulation, because they serve as specific recognition sites for many enzymes. JMJD2A-tudor domains are employed in the recognition of the specific methylation marks on H3 and H4 tails. Hence, JMJD2A enzyme is directed to specific locations on histone to function as a histone lysine demethylase. To understand the underlying reason of the varying binding affinities and the specificity towards different methylation patterns one has to carefully analyze structural and dynamical properties of the binding of these domains to the histone tails. In this manuscript we explain various aspects of the recognition by the tandem tudor domains and in this section we present a brief discussion.

As mentioned previously, JMJD2A-tudor recognizes and binds to four different methylated peptides: H3K4me3, H3K9me3, H4K20me2 and H4K20me3. The methylated peptides adopt two different binding modes of which one is adopted by H4 peptides and the other by H3 peptides. As expected, H4 peptides adopt the same binding mode since they share the same amino acid sequence. The only difference is that the H4 peptides have different number of methyl groups on Lys20. We found that removal of one methyl group from the trimethylated H4 peptide did not change the binding mode and most of the interactions with the receptor molecule. When we analysed the binding modes of the H3 peptides, we observed not only that the peptides adopt a similar binding mode but also that they form similar interactions with several receptor residues. Ser938 and Asn940 are involved in strong interactions with Thr3 and Arg8 of H3K4me3 and H3K9me3 peptides respectively. Likewise, Asp945 interacts with Arg2 and Arg8 of the peptides. Interactions with Asn940 and Ser938 suggest that Arg8 of H3K9me3 has a similar binding fashion with Thr3 of H3K4me3. Furthermore, electrostatic interactions between the trimethyllysine residues of H3K4me3 and H3K9me3 peptides and the Asp934 residue in the JMJD2A-tudor support the similarity of the recognition of these two peptides by the receptor.

To compare the recognition of tri- and dimethylated peptides we investigated the binding site differences between H4K20me3 and H4K20me2 ligands complexed with the tudor domains. Comparison of the conformational changes in the trimethyl and the dimethyl groups showed that the trimethyl groups continuously rotate, whereas the dimethyl group keeps its more stable conformation. To figure out the underlying basis for the varying stability, we investigated the overall changes in the neighbourhood of the methylated residues in detail. Suggested by the binding free energy differences, absence of the third methyl group in the H4K20 dimethylated lysine residue leads to strengthening of its interactions with the residues in the vicinity of the binding pocket. That increases the energy barriers for the g^+^→g^−^ and g^−^→g^+^ transitions [see Table 1]. The trimethyl residue, on the other hand, is subjected to lower energy barriers and therefore transitions between all states take place more frequently.

In this study we discovered the order of the binding affinities as H4K20me2 > H4K20me3 > H3K4me3 > H3K9me3, suggested by the binding free energies [Table 5]. The same order is obtained when the enthalpic values are compared. We see that H4K20me2 peptide forms the strongest interactions with the JMJD2A-tudor, thus result a larger enthalpic value. As discussed above, third methyl group in H4K20me3 decrease the strength of the interactions in the binding site. Therefore, enthalpy of JMJD2A-tudor-H4K20me3 structure shows up with a smaller value. Compared to that of H4 peptides, H3 peptides have lower binding affinities. Looking at the energetic values, one can see that H4 peptides form stronger electrostatic and van der Waals interactions. Many positively charged residues on the H4 peptides bind very tightly to the negatively charged surface of the HTD-2. Besides, the coulombic interactions between the ligands and the receptors result in stronger van der Waals interactions upon binding. Moreover, complexations of H3 peptides with the tudor domains result in internal strains hence result in high positive energetic contributions to the enthalpy.

Inspecting the energetic contributors [Table 3] from the PBSA calculations gives an insight in the binding differences. Nonpolar desolvation terms are negative for all complexes, showing that they are favourable components of the binding. The desolvation term is computed from the solvent accessible surface area and the protein itself assumed to be hydrophobic on the surface to obtain this term. Therefore, we obtain more or less similar favourable contributors to the binding free energy. On the other hand, polar desolvation term is a penalty term in the binding free energy and somewhat comparable with coulombic interaction energy. In our calculations, the polar desolvation terms compensate the MM electrostatic terms and the electrostatic contributors mostly diminish. However, trimethylated H4K20 has a large desolvation term compared to its coulombic term and this result in a highly unfavourable electrostatic contribution to the total binding free energy difference. The large desolvation energy leans to the fact that the protein and the ligand do not form sufficiently strong interactions upon binding to completely pay for the desolvation penalty. Nevertheless, resulting binding free energy difference is highly favourable, driven mostly by van der Waals interactions.

Ranking of the enthalpic differences upon binding is also consistent with the rankings of the energetic barriers of the conformational changes in the trimethyl and the dimethyl groups, hence proposing that binding affinities are positively correlated with the activation energies of conformational transitions. Comparison of the conformational changes also suggests that the trimethyl groups increase the entropy of the system [see Table 4] more than the dimethyl group. On the other hand, entropic contribution to the binding free energy of JMJD2A-tudor-H4K20me2 structure is larger than that of the structure with trimethylated K20. The difference in the entropy values mostly arise from the vibrational term of the entropy. RMSF values (Figure S4) show that overall the trimethylated structure is more stable compared to the dimethylated one. Strengthening of the interactions between the dimethyl group and the residues in the vicinity may lead to a slight decrease in the overall stability. Therefore, the resulting large entropic term is not surprising.

As mentioned in results section, the total enthalpic values were decomposed into residual components with each of the energetic contribution to the enthalpies. The energetic investigations of the four structures suggested that many of the residues involved in the interactions with the peptide ligands were common among the peptides. In this study, however three receptor residues that were related to selective binding of the H3 and H4 ligands: Asn940 was found to be important for the recognition of the H3 tails but not the H4 tails, whereas Asp939 and less significantly Glu929 was found to be important for the recognition of the H4 tails but not the H3 tails. Modifications or mutations on one of these residues can selectively alter the recognition of the H3 tails or the H4 tails by favouring or disfavouring.

## Materials and Methods

In this study, we performed 25ns fully unrestrained molecular dynamics simulations of the tudor domains of JMJD2A complexed with H3K4me3, H4K20me3, H4K20me2 and H3K9me3 histone tails along with the free structures. For the non standard trimethyllysine and dimethyllysine residues, parameters compatible with the Duan et al. force field were generated using quantum mechanical techniques. Docking simulations were carried out for JMJD2A-tudor-H3K9me3 complex before the simulations of the structure, since there was no available initial structure determined by the experiments. 2400 snapshots were extracted from the last 24 ns of the simulations with equally spaced 10 ps time intervals. Utilizing the snapshots, we calculated the enthalpic contributions to the binding free energies of four of the complexed structures conducting the three trajectory MM-GBSA approach (one for the complex, and two for the free proteins). Binding free energies were obtained after the removal of the entropic terms obtained by the NMODE calculations. Entropic and enthalpic contributions to the binding free energies were decomposed into their constituents. The residues having a contribution less than −1 kcal/mol to the free energy of binding were defined as hotspots.

### Preparation of initial coordinate files

Currently, there are 3 known crystal structures of JMJD2A-tudor: 2QQR [16] at 1.80 Å resolution, 2QQS[16] at 2.82 Å resolution and 2GFA [17] at 2.10 Å resolution. The 2QQR structure contains the tudor domains with no missing residues. For the initial coordinates, the B chain was selected from 2QQR. The 2QQS structure contains the tudor domains and H4K20me3 peptide together in bound form. The 2GFA structure contains the tudor domains and H3K4me3 peptide together in bound form. Both 2QQS and 2GFA structures have missing residues. B and D chains were selected for both 2GFA and 2QQS structures, respectively because the number of missing residues were less than those of the A and C chains. The rest of the missing residues in the tudor protein were completed by using SWISS MODELLER [25] homology modelling server. Each of the histone peptides in 2GFA and 2QQS structures consists of seven residues. In structure determination experiments, three residues could not be not located (Arg8, Lys9 and Ser10 in 2GFA and Lys16, Asp24 and Asn25 in 2QQS). These residues are at the terminals of the peptides.Since these missing residues were not reported to be significant in binding they left unmodelled in our simulations.

Initial JMJD2A-tudor-H4K20me3 structure was also used for the dimethylated state (H4K20me2) of the same complex. Since the structure of JMJD2A-tudor-H3K9me3 complex was not available, initial structure was modelled employing molecular docking simulations as explained below. Parameters for trimethyllysine and dimethyllysine residues in the peptide ligand were missing and parameterization was needed. New parameters for the non-standard residue were generated using quantum mechanical techniques (See below).

### Parameterization of non standard residues

To be consistent with the parameter set of the rest of the system, which was generated using the ff03 (Duan et al.) force field, [26] an initial parameterization procedure was carried out using quantum mechanical methods. For this purpose, initial coordinates of the non standard residues were generated as peptide fragments made up of Ace-trimethyllysine-Nme and Ace-dimethyllysine-Nme for the trimethylated and for the dimethylated residues respectively. For accuracy, two different conformations were used for the peptide fragments. The first one was the alpha conformation where dihedral angles were Φ, Ψ = −60, −40 respectively and the second one was the beta conformation where dihedral angles were Φ, Ψ = −120, 140 respectively. All of the three dimensional coordinates were obtained using Discovery Studio [27] (Accelrys Inc.).

Geometry optimization was done at the level of Restricted Hartree-Fock (RHF) theory with 6-31G* basis set. Multiplicity value and the total charge of the peptide were introduced as 0 and +1 respectively. Dihedral angles were fixed to their initial conformational states for geometry optimization. All quantum mechanical calculations were performed using Gaussian 03 [28] program.

After completing geometry optimization, molecular electrostatic potential calculation and RESP[29] fitting procedure were performed using Gaussian 03 and R.E.D. III[30] programs. Chemically equivalent methyl groups were set to have the same effective charges, and the total effective charges for acetyl and methyl caps were set to 0. Molecular electrostatic potential calculations were done using DFT, at the level of B3LYP theory with ccpVTZ basis set. IEFPCM was chosen as continuum solvent model and ether, which has a dielectric constant of 4, was chosen as the organic solvent which IEFPCM applied in.

Library files for the non standard residues were created using Leap which is an AMBER[31] tool. Atom types in the non standard residues were adapted from the general AMBER force field.

### Molecular Docking

To predict the binding mode of the complex, molecular docking simulations were performed with AutoDock 4.0[32], [33] docking tool. The structure of the receptor was taken from the initial structure of the unliganded tudor, whereas for the ligand, the G chain of the crystal structure 2Q8C[8] at 2.05 Å resolution, was used. The receptor and the ligand structures were then minimized 10,000 times separately using AMBER Sander in three different conditions: in vacuum, in implicit solvent and in explicit solvent. Following the minimizations, to obtain all possible binding modes, the input files for rigid and flexible docking simulations were prepared using AutoDock Tools 1.5.2[34] with the addition of Gasteiger[35] charges. HTD-2 of JMJD2A-tudor was selected to be accessible for the ligand, since we assumed that H3K9me3 tail would also be recognised by the same region as the other histone ligand complexes of JMJD2A-tudor. Finally, with the following properties, each of the docking simulations were performed for 100 runs: Lamarckian Genetic algorithm[32] as the searching algorithm, 25,000,000 number of evaluations, population size of 250, 50,000 number of generations with the rates of 0.8 and 0.02 for mutation and for crossover respectively. Owing to the computational expense of the simulations, only one binding mode with the lowest scoring function was selected for the molecular dynamics simulations. To test the accuracy of the initial docking conformation, we performed docking simulations of the crystal structures with the same docking criteria. We saw that Autodock reproduced docking conformations close to crystal structures if the binding site were chosen to be the searching region.

### MD Simulations

The NAMD[36] 2.5 molecular dynamics simulations package was used for all of the simulations for equilibrium and minimization steps as well as the production step. Because of the accuracy of the effective charges and the rest of the parameters, the Amber ff03[26] force field was selected for the molecular dynamics simulations. Minimization was carried out 25,000 times with a conjugate gradient method implemented in the NAMD.

Periodic boundary conditions were applied for equilibration and production run periods of the systems as in our previous studies [37], [38], [39]. The SETTLE[40] algorithm was used for keeping the bond lengths fixed in water molecules with a rigid bond tolerance of 10^−5^ Å. For full electrostatic interactions the Particle Mesh Ewald[41] (PME) regime was used since the interactions in periodic boundary conditions are extravagant. For the Lennard Jones interactions, a distance of 10 Å was used as the cutoff value. Coordinates and energies were collected at every 1ps where integration times of the simulations were chosen as 2fs. Systems were gradually annealed from 10 K to 310 K in a time period of 1500 ps. When the temperatures reached 310 K, the temperature was maintained using a Langevin thermostat with a coupling coefficient of 5/ps. Langevin dynamics were turned off for hydrogen atoms in the system. Since the simulations were performed in the isobaric-isothermal ensemble (NPT), constant pressure control was applied to the systems. Maintenance of the pressure at 1.01325 bar was carried out on the basis of Langevin piston Nose-Hoover[42], [43] method with a barostat oscillation time of 100 fs, a barostat damping time of 50 fs and a barostat noise temperature of 310 K. 50 ps of equilibration period were performed for each system after minimization and annealing steps.

The production simulations of the systems were performed for 25 ns using the methods as in the equilibration period. Coordinates and energy values were collected every 1 ps throughout the simulations.

### Binding Free Energy Calculations

The non covalent association of a receptor molecule and a ligand molecule in a solution is as follow:
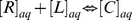
(1)where R stands for receptor, L stands for ligand and C stands for the complex that the receptor and the ligand form together. The association of the molecules generate a free energy difference that is related to the binding free energy of the ligand.The binding free energy of the ligand is also computed as the free energy difference between complex and receptor and ligand:
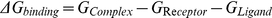
(2)


The binding free energy is formed of enthalpic end entropic contributions:
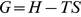
(3)where H is the enthalpy, T is the temperature and S is the entropy of the molecule. The Enthalpy of each of the molecules given in equation 3 is composed of two components: solute effect and the solvent effect to the free energy. To see the solute and solvent contributions, free energy may be restated as:
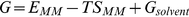
(4)where the first term E_MM_ is the average energy of the solute and comes from the bonded and non bonded molecular mechanics interactions:
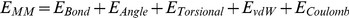
(5)where E_bond_ E_Angle_ and E_Torsional_ contributions stand for bonded interactions and E_vdW_ and E_Coulomb_ stand for non bonded interactions. In a computer simulation these contributions are obtained from molecular dynamics simulations.

The second term TS_MM_ in equation 4 comes from the entropic contribution of the solute. T represents temperature and S_MM_ represents the entropy that is obtained from molecular mechanics. In detail S_MM_ consists of the following terms: 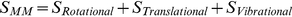
(6)where S_Rotational_, S_Translational_ and S_Vibrational_ stand for rotational, translational and vibrational motions of the solute, respectively.

The last term G_solvent_ in equation 4 comes from the solvent contribution to free energy and is composed of two components:
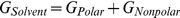
(7)where G_Polar_ stand for the polar contribution and is computed via Generalized Born (GB) method in this study. The second term G_Nonpolar_ stands for the nonpolar contribution and is computed from solvent accessible surface area (SASA):
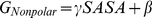
(8)where γ stands for surface tension, SASA stands for the solvent accessible surface area of the solute and β stands for an offset value.

### Activation Energy Calculations

To obtain activation energy of conformation transition, we utilized the Arrhenius equation:
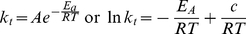
(9)where k_t_ is the transition rates between each states, A is temperature dependent constant and E_a_ is the activation energy of conformational transition. Since we do not have any experimental data of A, we simply take 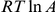 to be equal to some constant c.

To calculate the transition rates between the conformational states for each of the conformational transition, conformational states of the methyl groups were identified for each of the system. These conformational states were found by carrying out torsional angle analysis of the methyl groups in the methylated lysine residues. C_δ_, C_ε_, N_ζ_ and CZ atoms of the methyl groups were selected to compute the time evolutions of the torsional angles.

### MM-PBSA/GBSA and Normal Mode Calculations

Enthalpic calculations were performed using 2400 snapshots from the last 24 ns of the molecular dynamics simulations with 10 ps time intervals. PB calculations were carried out in DelPhi:[44] a finite difference Poisson-Boltzmann solver program. Parse radii[45] and Duan et al. charges were employed and the modified Bondi radii[46] were augmented by 1.4Å for PB calculations. GB calculations were carried out in MM-GBSA tool available in the AMBER 10 suite using the GB solver. The modified GB model[46], which was proposed by Onufriev et al., was selected for calculations. Both PB and GB calculations were performed for each structure based on internal dielectric constant 4 for protein and external dielectric constant 80 for solvent. For the SASA calculations the Molsurf[47] program, which is a part of AMBER simulation package, was used with the LCPO[48] method. To compute the nonpolar contributions to PBSA, γ and β values were taken as 0.00542 and 0.92 respectively, whereas for contributions to GBSA, γ and β values were taken as 0.005 and 0.0 respectively.

To find the hotspots of the protein, pair wise per residue free energy decomposition calculations were performed in the AMBER MM-PBSA tool using the GB model. Since the decomposition calculations work only with the ICOSA method, for the SASA calculations the ICOSA method was utilized.

Normal mode calculations were carried out in the AMBER NMODE module to find the entropic contributions of association. Because of the computational expense of the NMODE calculations, 240 snapshots were used from the last 24 ns of the molecular dynamics simulations with 100 ps time intervals. The calculations were performed using a distance dependent dielectric constant ε = 4R_ij_, that was applied for each structure.

Finally, to assess the convergence of the time evolutions of the values obtained from the MM-GBSA and the NMODE methods, mean and standard error of the values were computed. Shown in Figure S5, well convergence values were achieved for all systems.

## Supporting Information

Figure S1 Radius of gyration of the proteins versus time for each complex structure and for the receptor structure.
(0.07 MB DOC)

Figure S2 Root mean square deviations (RMSDs) for the complexed structures versus time. RMSDs were computed for each of the Hybrid Tudor Domain 1 (HTD-1), Hybrid Tudor Domain 2 (HTD-2) and histone tail along with the overall structures.
(0.17 MB DOC)

Figure S3 Distance between the center of the protein and the tip of the flap region. Center of the protein was chosen as the Ca of Val972 and the flap region point was chosen as the Ca of Pro982.
(0.03 MB DOC)

Figure S4 Root mean square fluctuations of the C, N and Cα atoms of the complex structures and the unliganded structure versus residue number in the structure.
(0.05 MB DOC)

Figure S5 Convergence of the mean values of the PB enthalpies and convergence of the standard errors of the PB enthalpies.
(0.06 MB DOC)
