# Supplementary figures and images for: Molecular Recognition of H3/H4 Histone Tails by the Tudor Domains of JMJD2A: A Comparative Molecular Dynamics Simulations Study
Source: PLoS One. 2011 Mar 25;6(3):e14765. doi: 10.1371/journal.pone.0014765 (PMC3064570; doi:10.1371/journal.pone.0014765)

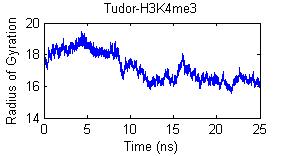

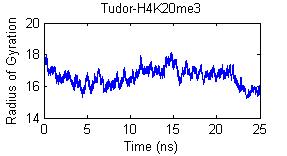


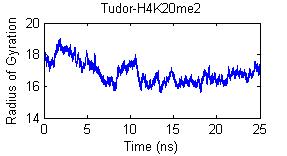

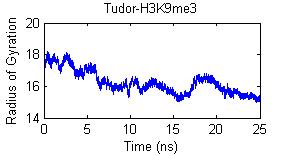


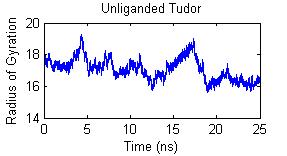

Supplement: Figure S1 — Radius of gyration of the proteins versus time for each complex structure and for the receptor structure. (0.07 MB DOC) [file pone.0014765.s001.doc]

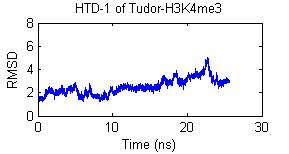

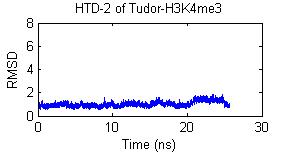

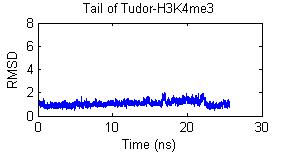

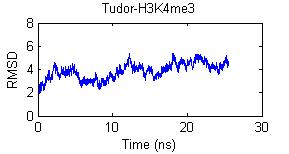


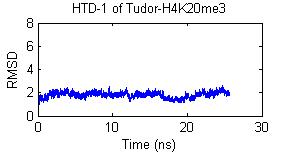

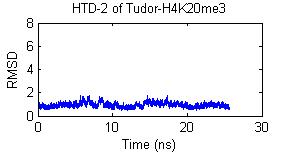

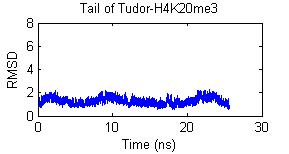

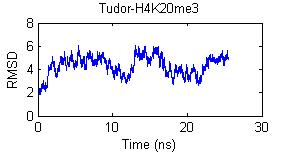


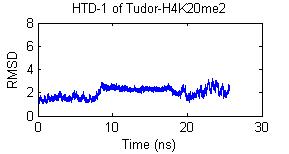

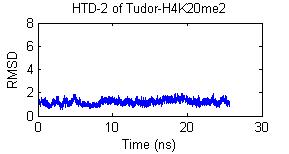

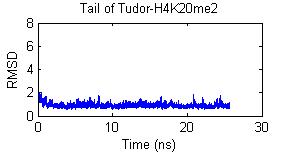

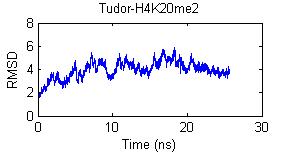


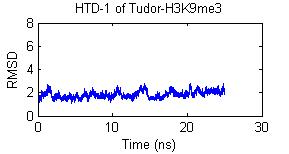

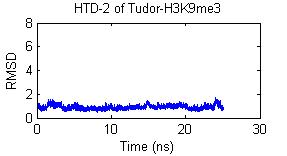

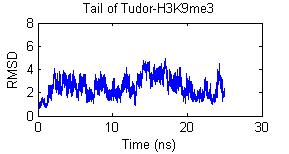

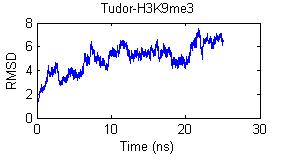

Supplement: Figure S2 — Root mean square deviations (RMSDs) for the complexed structures versus time. RMSDs were computed for each of the Hybrid Tudor Domain 1 (HTD-1), Hybrid Tudor Domain 2 (HTD-2) and histone tail along with the overall structures. (0.17 MB DOC) [file pone.0014765.s002.doc]

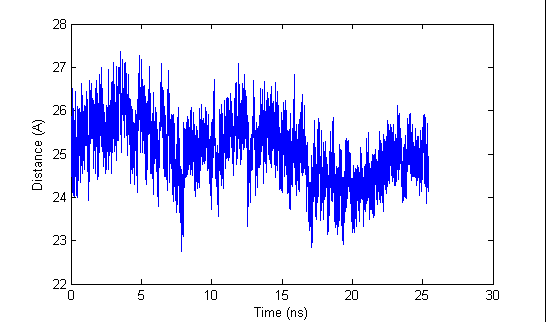

Supplement: Figure S3 — Distance between the center of the protein and the tip of the flap region. Center of the protein was chosen as the Ca of Val972 and the flap region point was chosen as the Ca of Pro982. (0.03 MB DOC) [file pone.0014765.s003.doc]

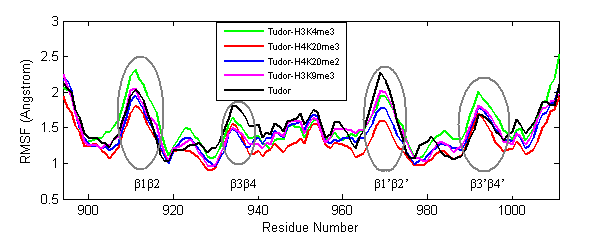


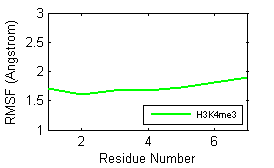

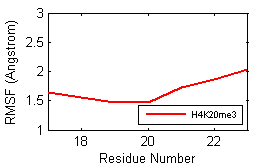


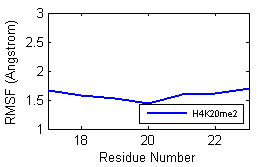

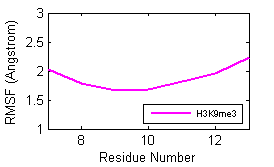

Supplement: Figure S4 — Root mean square fluctuations of the C, N and Cα atoms of the complex structures and the unliganded structure versus residue number in the structure. (0.05 MB DOC) [file pone.0014765.s004.doc]

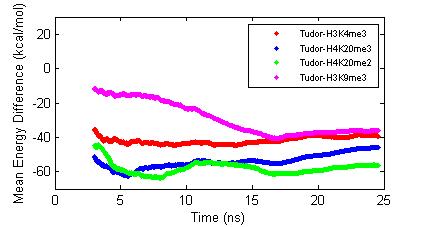

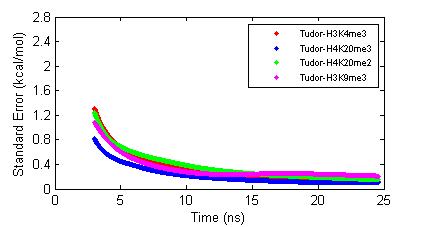

Supplement: Figure S5 — Convergence of the mean values of the PB enthalpies and convergence of the standard errors of the PB enthalpies. (0.06 MB DOC) [file pone.0014765.s005.doc]
